# Identification of TP53 mutations in circulating tumour DNA in high grade serous ovarian carcinoma using next generation sequencing technologies

**DOI:** 10.1038/s41598-023-27445-2

**Published:** 2023-01-06

**Authors:** Leslie Calapre, Tindaro Giardina, Aaron B. Beasley, Anna L. Reid, Colin Stewart, Benhur Amanuel, Tarek M. Meniawy, Elin S. Gray

**Affiliations:** 1 School of Medical and Health Sciences, Edith Cowan University, Joondalup, WA Australia; 2 Anatomical Pathology, PathWest Laboratory Medicine, QEII Medical Centre, Nedlands, WA Australia; 3 Centre for Precision Health, Edith Cowan University, Joondalup, WA Australia; 4 Medical School, University of Western Australia, Crawley, WA Australia; 5 Department of Medical Oncology, Sir Charles Gairdner Hospital, Nedlands, WA Australia

**Keywords:** Ovarian cancer, Genetics, Molecular biology, Cancer, Cancer genetics, Tumour biomarkers

## Abstract

Plasma circulating tumour DNA (ctDNA) has been suggested to be a viable biomarker of response to treatment in patients with high grade serous ovarian carcinoma (HGSOC). *TP53* mutations are present in more than 90% of HGSOCs but somatic variants are distributed across all exonic regions of the gene, requiring next generation sequencing (NGS) technologies for mutational analysis. In this study, we compared the suitability of the Accel (Swift) and Oncomine (ThermoFisher) panels for identification of *TP53* mutations in ctDNA of HGSOC patients (N = 10). Only 6 patients (60%) were found to have TP53 mutations using the ACCEL panel but the addition of molecular tags in the Oncomine panel improved ctDNA detection with at least one mutation detected in all cases (100%). Orthogonal validation of the 14 somatic variants found by Oncomine, using droplet digital PCR, confirmed 79% (11/14) of the identified mutations. Overall, the Oncomine panel with unique molecular identifiers (UMI) appears more useful for ctDNA analysis in HGSOC.

## Introduction

High-grade serous ovarian carcinoma (HGSOC) is the most common subtype of primary tubo-ovarian malignancy, accounting for approximately 70% of cases overall. Most cases present at advanced stage with peritoneal spread and often lymph node and/or distant metastasis. The serum glycoprotein cancer antigen 125 (CA-125) is a commonly used diagnostics biomarker in the investigation of patients presenting with possible ovarian neoplasia and for monitoring treatment response^1,2^. However, while CA-125 can indicate the trend of treatment response, it does not directly reflect absolute tumour volume^3^. Furthermore, it is limited by specificity, intra-tumoural and inter-patient heterogeneity and a long biological half-life in serum. Thus, a more sensitive and specific biomarker would be helpful in the management of HGSOC patients.

Recent studies have suggested that serum DNA-based biomarkers may have superior capabilities for reflecting tumour burden and measuring treatment response in cancer patients. In contrast to protein biomarkers, which typically are not specific to cancer cells, circulating tumour DNA (ctDNA) measures levels of mutations in plasma cell-free DNA, providing cancer-specific information. Plasma ctDNA fragments have a short half-life, and their levels have been shown in other cancer types to be related to tumour volume and response to treatment^4,5^. Thus, ctDNA appears to be a potentially useful adjunct biomarker in oncology.

More than 90% of patients with HGSOC have been shown to harbour mutations in the *TP53* gene^6–8^. Earlier studies have shown that *TP53* mutations can be detected in ctDNA from patients with advanced stage HGSOC and that, in a small number of patients studied, changes in ctDNA levels correlated with clinical response and outperformed CA-125^9–12^. However, *TP53* mutations in HGSOC are randomly distributed in all coding regions and chemotherapy has been shown to impose clonal evolution of *TP53* mutations; the resulting potential contraction or expansion of mutant clones therefore poses a challenge for patient surveillance during treatment. Thus, ctDNA analysis in HGSOC is likely to require next generation sequencing (NGS) technologies.

In this study, we directly compared two NGS platforms to identify *TP53* mutations in plasma ctDNA of HGSOC patients. We demonstrate the impact of unique molecular identifiers (UMI) and an efficient bioinformatics pipeline for improved sensitivity of NGS-based *TP53* profiling.

## Materials and methods

### Patients

We collected blood samples from 10 stage III and IV HGSOC patients with active disease enrolled for study in 2018 at Western Oncology Clinic and Sir Charles Gairdner Hospital (SCGH) in Perth, Western Australia. Written informed consent was obtained from all patients, and procedures were approved by the Human Research Ethics Committees from Edith Cowan University (No. 11543) and Sir Charles Gairdner Hospital (No. 2013-246) in compliance with Helsinki Declaration. Experiments were conducted per institutional and national guidelines and regulations.

### Tissue analysis

Tumour tissue from primary or interval debulking surgery was used for sequence analysis. Hematoxylin and eosin (H&E)-stained sections were assessed by a pathologist and the percentage of tumour cells estimated. Microdissection was performed when the neoplastic cell content was below 50%. DNA was isolated using QIAamp Tissue Formalin-Fixed Paraffin-Embedded (FFPE) Kits (Qiagen, Hilden, Germany) as per the manufacturer’s instructions. FFPE genomic DNA (gDNA) was stored at 4 °C until processed for targeted sequencing.

### Plasma samples preparation and cell-free DNA (cfDNA) extractions

Blood samples from stage four HGSOC were collected in EDTA vacutainer or Cell-Free DNA BCT (Streck, La Vista, NE) tubes and stored at 4 °C. Plasma was separated within 24 h by centrifugation at 300×*g* for 20 min, followed by a second centrifugation at 4700×*g* for 10 min and then stored at − 80 °C until extraction. cfDNA was isolated from 5 ml of plasma using QIAamp Circulating Nucleic Acid Kits (Qiagen, Hilden, Germany) as per the manufacturer’s instructions. Plasma cfDNA was eluted in 40 µl AVE buffer (Qiagen, Hilden, Germany) and stored at − 80 °C until analysis.

### Accel comprehensive TP53 assay library preparation and sequencing

DNA derived from FFPE tumour tissue (N = 10) and plasma samples (N = 10) underwent NGS. Matched non-neoplastic control tissue was examined in 5 patients to further delineate true somatic mutations from sequencing artefacts. DNA samples were amplified using a highly-multiplexed polymerase chain reaction (PCR) that amplifies 21 exonic regions of TP53 alone using the ACCEL-Amplicon TP53 Comprehensive Panel (Swift Biosciences). Forward and reverse strand NGS libraries were prepared according to the manufacturer’s instructions. In brief, forward and reverse oligonucleotide pools were hybridised to DNA samples overnight. Hybridised samples were then ligated, extended, and amplified with unique index sequences (barcodes) and sequencing adaptors. Amplified libraries were purified using Agencourt AMPure XP magnetic beads (Beckman Coulter, Brea, CA, USA), first at a ratio of 1 (library):1 (beads), as per the manufacturer’s protocol, followed by a second round of purification at a library to bead ratio of 1.25:1. Library DNA concentrations were quantified using a Qubit 3.0 fluorometer. Libraries were normalized to 4 nmol/l in EBT buffer, pooled, and sequenced on a MiSeq instrument (Illumina, San Diego, CA, USA). Sequence alignment and variant calling was performed using the Erase-Seq bioinformatics pipeline through collaboration with Fluxion. Variants with allele frequency (VAF) > 3% and ≥ 0.2% in tissue and cfDNA respectively were considered true mutations.

### Oncomine assay library preparation, sequencing and bioinformatics

Plasma cfDNA derived from 10 plasma samples underwent library preparation. DNA samples were amplified using highly multiplexed PCR that amplifies 17 exonic regions of *TP53* and 135 hotspots in nine genes (*AKT1*, *EGFR*, *ERBB2*, *ERBB3*, *ESR1*, *FBXW7*, *KRAS*, *PIK3CA*, *SF3B1*), and copy numbers in three genes (*CCND1*, *ERBB2*, *FGFR1*) commonly mutated in breast cancer. Sequencing libraries were created using the Oncomine Breast cfDNA v2 assay (Thermo Fisher), subjected to emulsion PCR, and sequencing templates were prepared in the Ion Chef (Thermo Fisher) using the Ion 530 Kit-Chef (Thermo Fisher). Five different barcoded libraries were pooled and loaded onto one Ion 530 chip (Thermo Fisher) to obtain appropriate coverage. Sequencing was carried out using an Ion S5 sequencer (Thermo Fisher). Each Ion 530 chip produced approximately 18 million reads. Reads underwent primary analysis using the Ion Reporter 5.10.5.0, which includes quality control, read trimming, and mapping to the human genome (hg19). The threshold for identification of true somatic variants was based on unique molecular identifiers (UMI). Variants are considered true mutations if they have a total molecular coverage ≥ 1000 and allele molecular coverage (AMC) of ≥ 1.

### Droplet digital PCR (ddPCR)

Commercially available and/or customized *TP53* probes were used to analyse ctDNA by ddPCR. Droplets were generated using an Automatic Droplet generator QX200 AutoDG (Bio‐Rad, Hercules, CA). Amplifications were performed using cycling conditions previously described^13^.

### Statistics

Pearson correlation was employed to determine the correlation between allele molecular coverage (ONCOMINE) and copies per 20 µl reaction (ddPCR). Statistical analyses were performed using GraphPad Prism version 5.

## Results

### Identification of TP53 mutations in tissue

We first identified single nucleotide variations (SNVs) in *TP53* from tumour tissue. The mutations identified in the tissue served as a guide for the identification of SNVs in plasma cfDNA. The ACCEL-Amplicon TP53 Comprehensive Panel (Accel) was used for mutational profiling in the tissue as it provided comprehensive exonic coverage of the *TP53* gene. Ten tissue samples with confirmed HGSOC were analysed. In addition, five adjacent samples of non-neoplastic tissue were included as negative controls. Each tissue sample in this study was sequenced to at least 1000× coverage, with a median coverage of 15,000× (range 1000×–25,000×). We found a total of 13 SNVs in *TP53* in 8 of the 10 tumours analysed (80%). The mutations and corresponding frequency abundance are summarised in Table S1.

### Comparison of NGS panel for TP53 analysis in ctDNA

We next compared the efficiency of the Accel panel to the Oncomine Breast cfDNA Assay V2 (Oncomine) for ctDNA detection in HGSOC. Comparison of the technical specification between Oncomine and Accel are detailed in Table S2. Mutations identified using these panels are also summarised in Table S1.

Using the Accel panel, plasma ctDNA was sequenced to a median coverage of 33,563× (Table S1), ranged between 1000 and 62,081× (Table S1). Nine SNVs were identified across 21 loci of TP53 (Table S1) in 6 out of 10 patients (60%), using the Erase-Seq bioinformatics pipeline. The pipeline has a limit of detection (LOD) of 0.2% and the fractional abundance (FA) of the mutations identified via the Accel NGS panel ranged from 0.2 to 3% (Table S1). Only three patients (OC1, OC4 and OC10) had one concordant mutation between plasma ctDNA and tissue biopsy. In the other 7 patients, TP53 mutations were found at relatively high frequency in plasma but these were not confirmed in the tissue (Fig. S2).

Overall sequence coverage using the Oncomine panel ranged between 21,021Xand 153,440×, with a median coverage of 69,773 (Table S1). In addition, the molecular coverage of each locus was ≥ 1000× in all plasma ctDNA analysed. A total of 21 SNVs were found in all 10 patients (Fig.S1), which ranged from 0.03 to 0.86% FA (Table 1). Out of the 21 SNVs identified in plasma, only four mutations; p.R282G, p.I195T, p.R248Q and p.R273C from OC1, OC3, OC4 and OC10 respectively, were also found in the tissue (Table S1). Three of these four mutations were also detected by the Accel panel in the plasma. Overall, there was a low concordance (28%) in the mutational profile obtained between plasma ctDNA and tissue. In one patient (OC7), the Oncomine panel also identified a *KRAS* p.G12D mutation in addition to *TP53* mutations. The *KRAS* gene is mutated in 14% of ovarian cancers, commonly in codon G12 (41%, COSMIC).

**Table 1:** Somatic mutations identified in ctDNA using the ONCOMINE and ACCEL NGS panels and validated by ddPCR.

Patient ID	Gene	Mutation	Chromosomal position	Oncomine panel	Accel comprehensive TP53 panel	ddPCR validation
OC1	*TP53*	p.R282G	17:7,577,094	**0.54%**	**0.3%**	**0.7%**
		p.G266R	17:7,577,142	*0.03%*^*a*^	*–*	*0.02%*
OC2	*TP53*	p.R273H	17:7,577,120	0.04%	–	–
OC3	*TP53*	p.I195T	17:7,578,265	*0.03%*^*a*^	*–*	*0.03%*
	*TP53*	p.N13N	17:7,579,757	^b^	0.2%	–
OC4	*TP53*	p.R282W	17:7,577,094	*0.10%*	*–*	*0.7%*
		p.R273L	17:7,577,120	0.10%	–	–
		p.R248Q	17:7,577,538	**0.86%**	**0.6%**	**0.50%**
		p.C238Y	17:7,577,568	*0.38%*	*–*	*0.10%*
		p.Y220C	17:7,578,190	*0.28%*	*–*	*0.25%*
		p.R280S	17:7,577,100	**2%**	**3%**	**3%**
OC5	*TP53*	p.T253P	17:7,577,524	–	0.3%	–
		p.R282W	17:7,577,094	*0.04%*	*–*	*0.04%*
		p.I195T	17:7,578,265	**0.86%**	**0.7%**	**0.83%**
OC6		p.G266R	17:7,577,142	*0.04%*	*–*	*0.11%*
OC7	*TP53*	p.S378fs	17:7,572,976	0.08%	–	^c^
OC8	*TP53*	p.R196Q	17:7,578,262	0.29%	–	^c^
		p.S185G	17:7,578,377	–	0.2%	–
		p.D184H	17:7,578,380	–	0.2%	–
OC9	*TP53*	p.Y234C	17:7,577,580	0.04%	–	–
OC10	*TP53*	p.R273C	17:7,577,121	**0.25%**	**0.27%**	**0.58%**

Bold denotes concordant variants identified using the Accel and Oncomine in cfDNA that were confirmed by ddPCR. Italics denotes variants found in Oncomine or Accell that are confirmed via orthogonal validation using ddPCR.

^a^Below threshold.

^b^Not covered.

^c^Not tested.

–, not detected.

**Figure 1:**
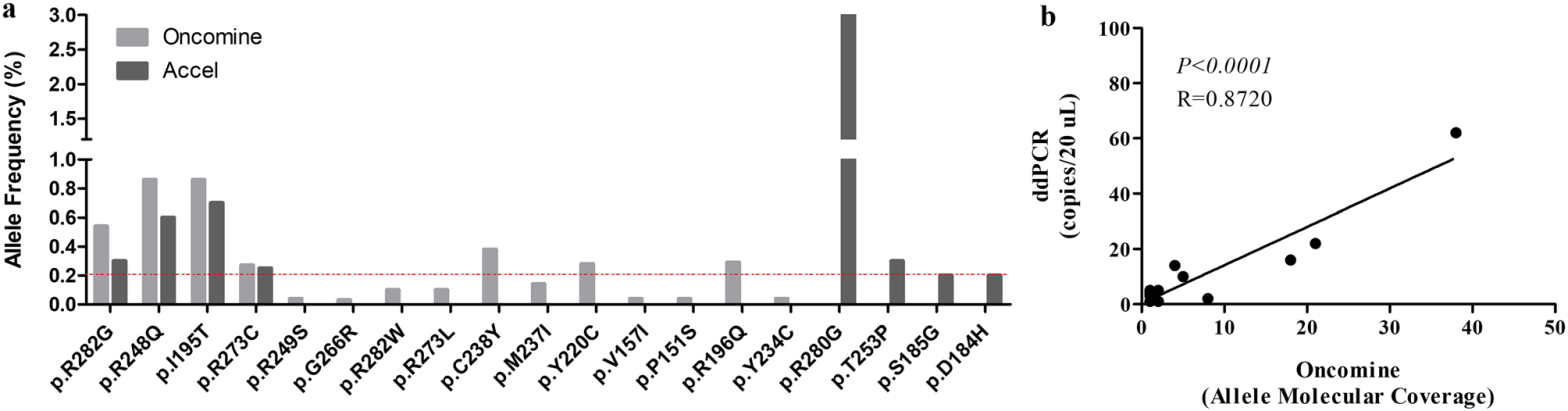
Comparison of SNVs identified via Accel vs Oncomine in the plasma of HGSCO patients. (**a**) Bar graph denotes the SNVs and corresponding allele frequency (%) that are found by both ONCOMINE and ACCEL, ONCOMINE alone or ACCEL alone. Red line denotes the limit of detection (LOD) for the Accel NGS panel. (**b**) Correlation of the number of mutant copies derived from NGS (Oncomine, UMI) and ddPCR. Analysis was performed using Pearson’s correlation and P < 0.05 is considered significant.

We also compared the mutations identified in ctDNA by Accel and Oncomine (Table 1). We found four TP53 mutations, which ranged from 0.2 to 0.8% allele frequency, in the plasma of 4 different HGSOC patients via the Accel and Oncomine sequencing panels (Fig. 1a). In addition, there were 11 plasma mutations (range: 0.03% to 0.86% FA), detected by the Oncomine panel but not with Accel. Of note, 9 of the 11 mutations were below 0.2% allele frequency, the analytical threshold of the Accel panel. By contrast, there were four mutations (p.R280S, p.T253P, p.S185G and p.D184H) that were found above 0.2% allele frequency by the Accel sequencing panel but were not confirmed in the same plasma sample by Oncomine despite sufficient total and molecular coverage. Lastly, the region covering a silent N13N mutation found in ctDNA by the Accel panel in OC3 is not covered by the Oncomine panel.

### Orthogonal validation of SNVs identified using Accel or Oncomine

We conducted orthogonal validation using ddPCR of 8 and 15 mutations identified by the Accel and Oncomine panels respectively, with four of these mutations found in both NGS platform. Only five of the 8 mutations (62.5%) identified by the Accel panel were confirmed by ddPCR, including the four mutations that were also detected by Oncomine (Table 1). By contrast, 12 of the 15 mutations (80%) found by Oncomine were confirmed by ddPCR, including 3 mutations that were deemed below the analytical threshold for Oncomine (0.03% FA).

Notably of the 12 mutations found by Oncomine and validated by ddPCR, the number of mutant copies found via NGS, which is indicated as the total AMC, was significantly concordant to the relative copies in the 8 µl of cfDNA added to the ddPCR reaction (R = 0.8720, P < 0.0001, Fig. 1b).

### TP53 mutations for ctDNA tracking

**Figure 2:**
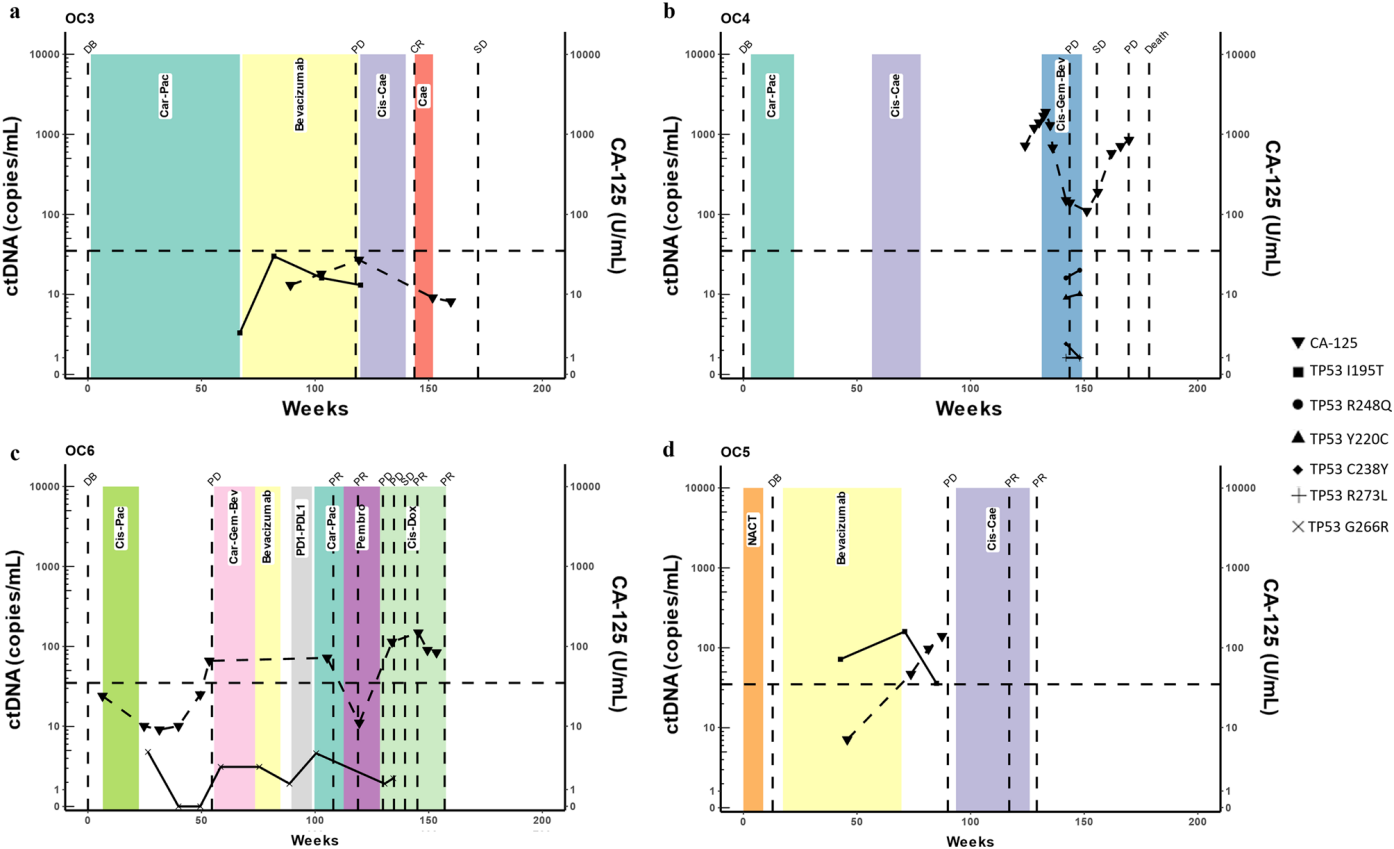
Longitudinal patient monitoring via *TP53* mutation in plasma ctDNA. (**a–d**) Line graph denotes the clinical status of four HGSOC patients that were longitudinally monitored throughout their disease course. ctDNA levels indicated by solid lines, different mutations indicated by changing point. CA-125 indicated by dashed line graph with upside-down triangles. Horizonal dashed line indicates the CA-125 positivity threshold of 35 units/mL of blood. Therapy time frames are indicated by coloured boxes with treatment noted in the centre. Vertical dashed lines indicate time of scans or surgery with the outcome named above the line. *PD* progressive disease, *SD* stable disease, *PR* partial response, *CR* complete response, *DB* debulking.

We used TP53 mutations identified via NGS to monitor response in four patients in this cohort for whom we collected longitudinal samples throughout their treatment course. Plasma ctDNA were quantified using ddPCR and represented in each patient graph as copies per ml of plasma. Three of the four patients (OC3, OC4 and OC6) had received adjuvant treatment approximately 1 week after interval debulking surgery, whilst OC5 had neoadjuvant chemotherapy prior to surgery (Fig. 2, Table S3).

For patient OC3 (Fig. 2a), sub-optimal debulking surgery led to > 2 cm residual disease and 12 months of maintenance with anti-VEGF therapy (Bevacizumab). Post-Bevacizumab the patient was well and without new symptoms from weeks 67–103, but increasing ctDNA levels eventually coincided with disease progression on radiological imaging (week 118). Similarly, patient OC4 (Fig. 2b), who had several rounds of chemotherapy had detectable ctDNA at week 142, which correlated with progressive disease in the pelvis, peritoneum and rectus abdominis that was confirmed by PET scan on week 144. Likewise, the longitudinal disease monitoring of patient OC6 (Fig. 2c) revealed detectable ctDNA levels post adjuvant chemotherapy, suggesting residual disease.

Patient OC5 (Fig. 2d) was treated with neoadjuvant chemotherapy prior to debulking surgery and maintenance Bevacizumab post-surgery. Detectable ctDNA levels between weeks 30–72 during maintenance anti-VEGF therapy appeared to indicate the presence of residual disease, culminating in the confirmation of disease progression in the peritoneum and lymph nodes at week 74. However, the decline in ctDNA at week 72, from week 36, was followed by apparent disease control by week 104, likely due to the Cisplatin/Doxil treatment.

## Discussion

Previous studies have shown that *TP53* mutations are a potentially useful target for ctDNA analysis in patients with advanced HGSOC^9–12^. However, somatic variants are distributed across all exonic regions of the gene. Thus, interrogation of the entire *TP53* gene is required for comprehensive and accurate ctDNA analysis in these tumours. Herein, we compared two NGS platforms that are suitable for *TP53* mutation identification in plasma ctDNA and demonstrated the impact of UMI for improved sensitivity of NGS-based *TP53* mutational profiling.

Overall, our results appear to indicate that the low LOD of both Oncomine and Accel panels makes them suitable for TP53 mutational profiling in ctDNA of HGSOC patients. All exonic regions of *TP53* are represented in the Accel panel. However, reduction of the threshold to 0.2% required the use of customised analysis pipelines such as ERASE-Seq. By contrast, the addition of molecular tags in the Oncomine panel improved *TP53* mutation detection allowing identification of SNVs at < 0.05% frequency abundance. However, only 80% of the exonic regions of *TP53* are covered and thus while the Oncomine panel appears to have superior detection sensitivity than the Accel, it precludes identification of potential clinically relevant mutations in unrepresented exonic regions. Furthermore, there are a few limitations with amplicon sequencing over hybrid capture. First, tiling amplicons in one pool can lead to interactions reducing quality or requiring non-tiled amplicons reducing coverage. Splitting primers into two pools would overcome this but may reduce sensitivity for rare variants as found in ctDNA. Furthermore, mutations in the primer region may cause dropout of the amplicon. However, amplicon sequencing has several benefits including reduced cost, easier automation, less hands-on-time, and by virtue of the method have higher on-target rates compared to hybrid capture.

Mutational profiling through the use of a liquid biopsy is challenging due to the mainly low amounts of circulating cfDNA and the very low representation of mutated cfDNA molecules extractable from the blood plasma. Therefore, methods which are able to detect a small number of mutated molecules in an abundance of wild-type DNA fragments with high sensitivity and specificity are required^14,15^. The addition of UMI, random nucleotide sequences barcoding each DNA-fragment prior to PCR amplification, has been demonstrated to overcome the drawbacks of PCR-based NGS, including DNA polymerase errors and generation of PCR duplicates as a result of sequencing the same molecule multiple times^16^. This has led to the discovery of mutations at < 0.1% FA which is within the typical range of ctDNA in plasma, especially in treated patients. Currently, quantitative PCR, amplification refractory mutation system (ARMS), digital PCR, beads, emulsion, amplification, and magnetics (BEAMing) and NGS are widely used^17^. While these methods enable a sensitive detection down to 0.01%, only NGS facilitates the parallel detection of a broader range of mutations by multi-gene or multi-target gene panels. In general, it appears as though ctDNA might be a better blood-based biomarker for monitoring patients compared to CA-125^18^. Similarly to our study, others have also utilised NGS with UMI barcoding and ddPCR for patient monitoring in HGSOC, similarly showing good correlations with changes in patient disease states^19^. In addition to mutation screening, other avenues of predicting patient response to therapy or disease progression may be through the use of somatic copy number alterations in ctDNA given the propensity of HGSOC to harbour them^20^.

One of the most critical aspects in analysing low VAF mutations in the plasma is the accumulation of somatic mutations to haematopoietic cells known as clonal haematopoiesis of indeterminate potential (CHIP), greatly increasing with age^21^. For example, the majority of healthy people aged over 50 have nonsynonymous mutations found both in matched cfDNA and blood cell DNA^22^, with *TP53* routinely detected as a CHIP-derived mutation^21–24^. This may be problematic in HGSOC ctDNA analysis due to the prevalence and non-hotspot nature of *TP53* mutations. Possibly future studies should concurrently sequence matched ctDNA and peripheral blood mononuclear cells to eliminate mutations associated with CHIP.

Previous studies have suggested that disease evolution impose somatic mutational landscape changes, which is an important consideration when selecting mutations for longitudinal patient monitoring. A significant caveat of this study was the long gap between blood collection and tumour tissue sampling in the majority of patients (Table 2). Thus, the discordance of the mutational profile may be attributed to clonal evolution in the intervening period between plasma ctDNA isolation and tissue biopsy. Other studies assessing NGS or digital PCR approaches for HGSOC prior to therapy have shown that ctDNA is readily detectable using TP53^25–27^, even as early as stage I^27^. Furthermore, ctDNA dynamics during treatment, particularly poor reduction of ctDNA levels, are capable of predicting worse outcomes^25,26^, indicating the clinical utility of ctDNA in HGSOC management.

**Table 2:** Blood collections and timings.

Patient	Stage	1st round chemotherapy (cycles)	Length of time^#^ (weeks)	Concordant mutations in blood vs tissue (%)	Blood tube	Time to process (HH:MM)	Volume (ml)	Nearest CA-125 level (U/ml)
OC1^	IIIa	Carboplatin/paclitaxel (1)	− 4	1 (50%)	EDTA	3:44	5	–
OC2	III	Carboplatin/paclitaxel (6)	− 14	0 (0%)	EDTA	5:11	5	–
OC3	IIIc	Carboplatin/paclitaxel (6)	− 67	1 (50%)	EDTA	20:34	5	13
OC4	IIIc	Carboplatin/paclitaxel (6)	− 142	1 (50%)	EDTA	3:12	5	142
OC5	IVa	Chemotherapy (Neoadj) (3)	− 30	N/A (CR*)	EDTA	4:21	5	33
OC6	III	Cisplatin/paclitaxel (6)	− 26	0 (0%)	EDTA	4:21	5	25
OC7	III	Carboplatin/paclitaxel (Neoadj) (3)	11	0 (0%)	EDTA	23:02	5	–
OC8	III	Cisplatin/pegylated liposomal doxorubicin (Neoadj) (4)	− 22	0 (0%)	Streck	19:15	4.5	–
OC9	III	Cisplatin/gemcitabine (6)	− 196	0 (0%)	EDTA	2:23	5	–
OC10	IIIc	Carboplatin/paclitaxel (6)	− 109	1 (100%)	EDTA	23:04	5	–

*N/A* not assessable.

^#^Time between blood collection and tissue biopsy.

*CR—no residual tumour tissue at interval debulking.

^Ascites noted after debulking.

Overall, our study demonstrates the utility of a UMI-tagged NGS panel for plasma *TP53* mutation screening in HGSOC patients. Further studies will need to address if this methodology will be suitable for measuring residual disease and response to therapy.

## Supplementary Information

Supplementary Information.

## Data Availability

Data available upon reasonable request to the corresponding authors, Elin Gray, e.gray@ecu.edu.au.
