# Supplementary material for: Identification of TP53 mutations in circulating tumour DNA in high grade serous ovarian carcinoma using next generation sequencing technologies
Source: Sci Rep. 2023 Jan 6;13:278. doi: 10.1038/s41598-023-27445-2 (PMC9822997; doi:10.1038/s41598-023-27445-2)
Supplement: Supplementary file 1 — Supplementary Information. [file 41598_2023_27445_MOESM1_ESM.pptx]

## Slide 1
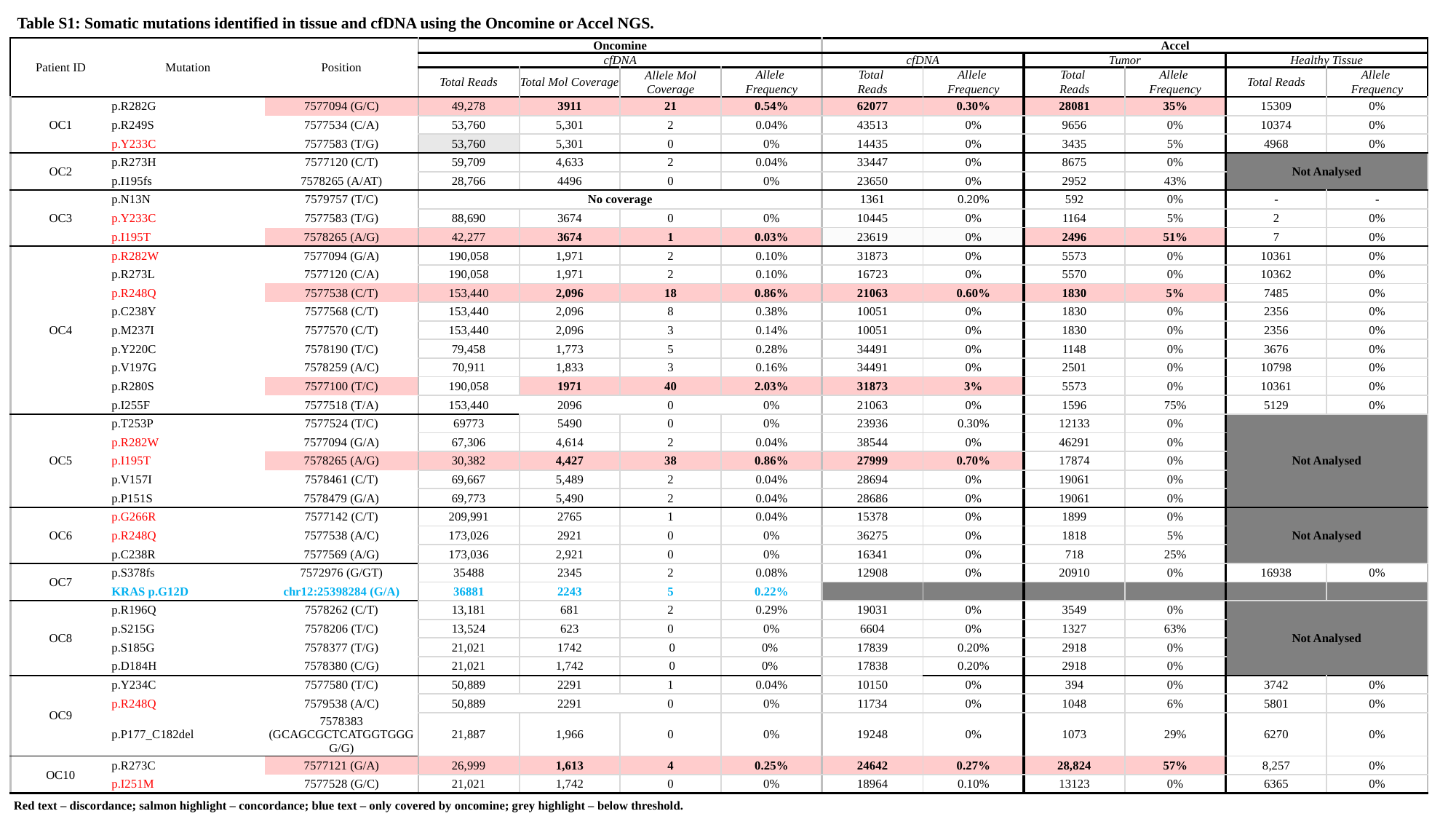

Table S1: Somatic mutations identified in tissue and cfDNA using the Oncomine or Accel NGS.
| Patient ID | Mutation | Position | Oncomine | | | | | Accel | | | | |
| --- | --- | --- | --- | --- | --- | --- | --- | --- | --- | --- | --- | --- |
| | | | cfDNA | | | | cfDNA | | Tumor | | Healthy Tissue | |
| | | | Total Reads | Total Mol Coverage | Allele Mol Coverage | Allele | Total | Allele | Total | Allele | Total Reads | Allele |
| | | | | | | Frequency | Reads | Frequency | Reads | Frequency | | Frequency |
| OC1 | p.R282G | 7577094 (G/C) | 49,278 | 3911 | 21 | 0.54% | 62077 | 0.30% | 28081 | 35% | 15309 | 0% |
| | p.R249S | 7577534 (C/A) | 53,760 | 5,301 | 2 | 0.04% | 43513 | 0% | 9656 | 0% | 10374 | 0% |
| | p.Y233C | 7577583 (T/G) | 53,760 | 5,301 | 0 | 0% | 14435 | 0% | 3435 | 5% | 4968 | 0% |
| OC2 | p.R273H | 7577120 (C/T) | 59,709 | 4,633 | 2 | 0.04% | 33447 | 0% | 8675 | 0% | Not Analysed | |
| | p.I195fs | 7578265 (A/AT) | 28,766 | 4496 | 0 | 0% | 23650 | 0% | 2952 | 43% | | |
| OC3 | p.N13N | 7579757 (T/C) | No coverage | | | | 1361 | 0.20% | 592 | 0% | - | - |
| | p.Y233C | 7577583 (T/G) | 88,690 | 3674 | 0 | 0% | 10445 | 0% | 1164 | 5% | 2 | 0% |
| | p.I195T | 7578265 (A/G) | 42,277 | 3674 | 1 | 0.03% | 23619 | 0% | 2496 | 51% | 7 | 0% |
| OC4 | p.R282W | 7577094 (G/A) | 190,058 | 1,971 | 2 | 0.10% | 31873 | 0% | 5573 | 0% | 10361 | 0% |
| | p.R273L | 7577120 (C/A) | 190,058 | 1,971 | 2 | 0.10% | 16723 | 0% | 5570 | 0% | 10362 | 0% |
| | p.R248Q | 7577538 (C/T) | 153,440 | 2,096 | 18 | 0.86% | 21063 | 0.60% | 1830 | 5% | 7485 | 0% |
| | p.C238Y | 7577568 (C/T) | 153,440 | 2,096 | 8 | 0.38% | 10051 | 0% | 1830 | 0% | 2356 | 0% |
| | p.M237I | 7577570 (C/T) | 153,440 | 2,096 | 3 | 0.14% | 10051 | 0% | 1830 | 0% | 2356 | 0% |
| | p.Y220C | 7578190 (T/C) | 79,458 | 1,773 | 5 | 0.28% | 34491 | 0% | 1148 | 0% | 3676 | 0% |
| | p.V197G | 7578259 (A/C) | 70,911 | 1,833 | 3 | 0.16% | 34491 | 0% | 2501 | 0% | 10798 | 0% |
| | p.R280S | 7577100 (T/C) | 190,058 | 1971 | 40 | 2.03% | 31873 | 3% | 5573 | 0% | 10361 | 0% |
| | p.I255F | 7577518 (T/A) | 153,440 | 2096 | 0 | 0% | 21063 | 0% | 1596 | 75% | 5129 | 0% |
| OC5 | p.T253P | 7577524 (T/C) | 69773 | 5490 | 0 | 0% | 23936 | 0.30% | 12133 | 0% | Not Analysed | |
| | p.R282W | 7577094 (G/A) | 67,306 | 4,614 | 2 | 0.04% | 38544 | 0% | 46291 | 0% | | |
| | p.I195T | 7578265 (A/G) | 30,382 | 4,427 | 38 | 0.86% | 27999 | 0.70% | 17874 | 0% | | |
| | p.V157I | 7578461 (C/T) | 69,667 | 5,489 | 2 | 0.04% | 28694 | 0% | 19061 | 0% | | |
| | p.P151S | 7578479 (G/A) | 69,773 | 5,490 | 2 | 0.04% | 28686 | 0% | 19061 | 0% | | |
| OC6 | p.G266R | 7577142 (C/T) | 209,991 | 2765 | 1 | 0.04% | 15378 | 0% | 1899 | 0% | Not Analysed | |
| | p.R248Q | 7577538 (A/C) | 173,026 | 2921 | 0 | 0% | 36275 | 0% | 1818 | 5% | | |
| | p.C238R | 7577569 (A/G) | 173,036 | 2,921 | 0 | 0% | 16341 | 0% | 718 | 25% | | |
| OC7 | p.S378fs | 7572976 (G/GT) | 35488 | 2345 | 2 | 0.08% | 12908 | 0% | 20910 | 0% | 16938 | 0% |
| | KRAS p.G12D | chr12:25398284 (G/A) | 36881 | 2243 | 5 | 0.22% | | | | | | |
| OC8 | p.R196Q | 7578262 (C/T) | 13,181 | 681 | 2 | 0.29% | 19031 | 0% | 3549 | 0% | Not Analysed | |
| | p.S215G | 7578206 (T/C) | 13,524 | 623 | 0 | 0% | 6604 | 0% | 1327 | 63% | | |
| | p.S185G | 7578377 (T/G) | 21,021 | 1742 | 0 | 0% | 17839 | 0.20% | 2918 | 0% | | |
| | p.D184H | 7578380 (C/G) | 21,021 | 1,742 | 0 | 0% | 17838 | 0.20% | 2918 | 0% | | |
| OC9 | p.Y234C | 7577580 (T/C) | 50,889 | 2291 | 1 | 0.04% | 10150 | 0% | 394 | 0% | 3742 | 0% |
| | p.R248Q | 7579538 (A/C) | 50,889 | 2291 | 0 | 0% | 11734 | 0% | 1048 | 6% | 5801 | 0% |
| | p.P177\_C182del | 7578383 (GCAGCGCTCATGGTGGGG/G) | 21,887 | 1,966 | 0 | 0% | 19248 | 0% | 1073 | 29% | 6270 | 0% |
| OC10 | p.R273C | 7577121 (G/A) | 26,999 | 1,613 | 4 | 0.25% | 24642 | 0.27% | 28,824 | 57% | 8,257 | 0% |
| | p.I251M | 7577528 (G/C) | 21,021 | 1,742 | 0 | 0% | 18964 | 0.10% | 13123 | 0% | 6365 | 0% |
| Red text – discordance; salmon highlight – concordance; blue text – only covered by oncomine; grey highlight – below threshold. | | | | | | | | | | | | |

## Slide 2
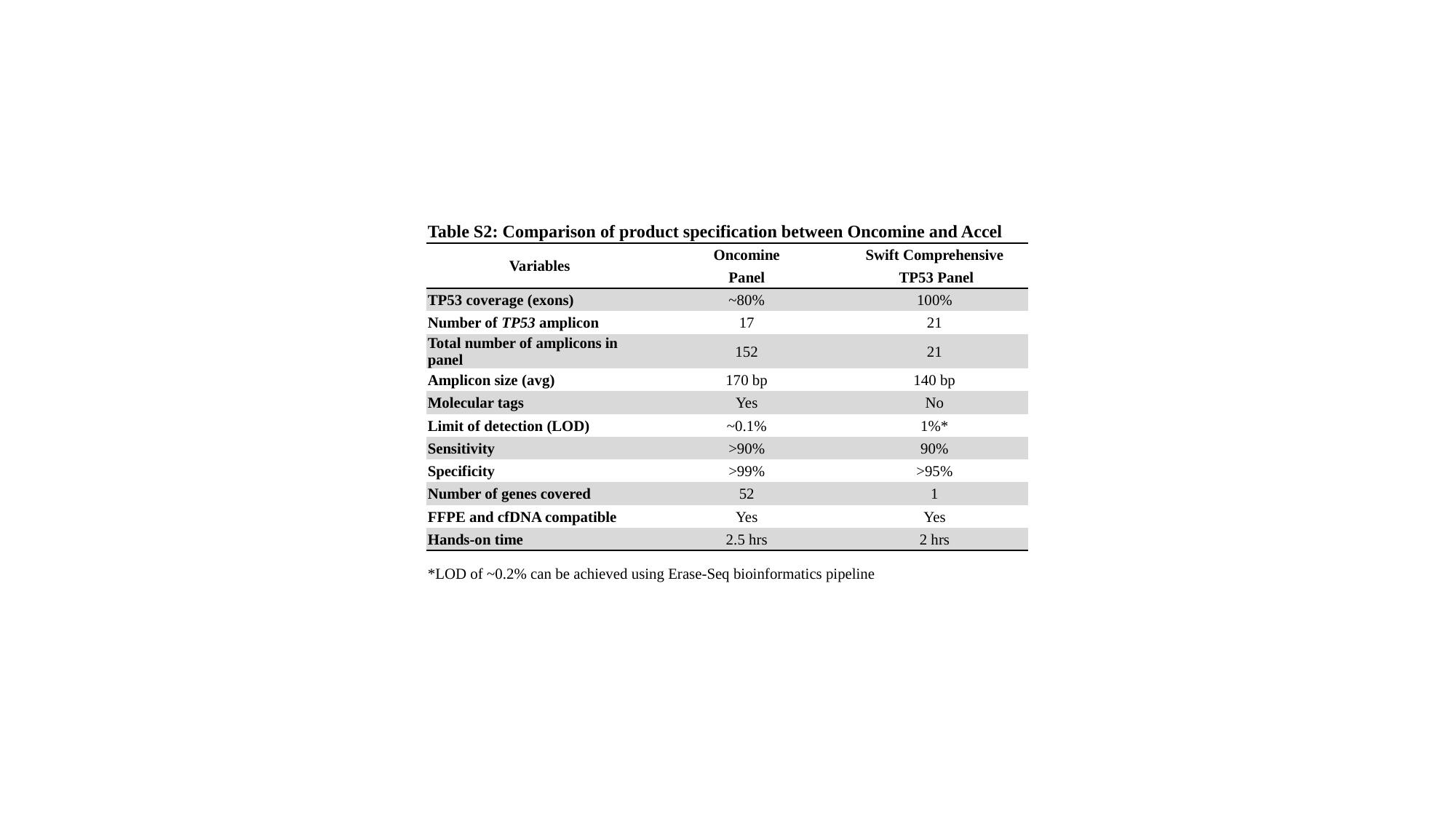

| Table S2: Comparison of product specification between Oncomine and Accel | | |
| --- | --- | --- |
| Variables | Oncomine | Swift Comprehensive |
| | Panel | TP53 Panel |
| TP53 coverage (exons) | ~80% | 100% |
| Number of TP53 amplicon | 17 | 21 |
| Total number of amplicons in panel | 152 | 21 |
| Amplicon size (avg) | 170 bp | 140 bp |
| Molecular tags | Yes | No |
| Limit of detection (LOD) | ~0.1% | 1%\* |
| Sensitivity | >90% | 90% |
| Specificity | >99% | >95% |
| Number of genes covered | 52 | 1 |
| FFPE and cfDNA compatible | Yes | Yes |
| Hands-on time | 2.5 hrs | 2 hrs |
| \*LOD of ~0.2% can be achieved using Erase-Seq bioinformatics pipeline | | |

## Slide 3
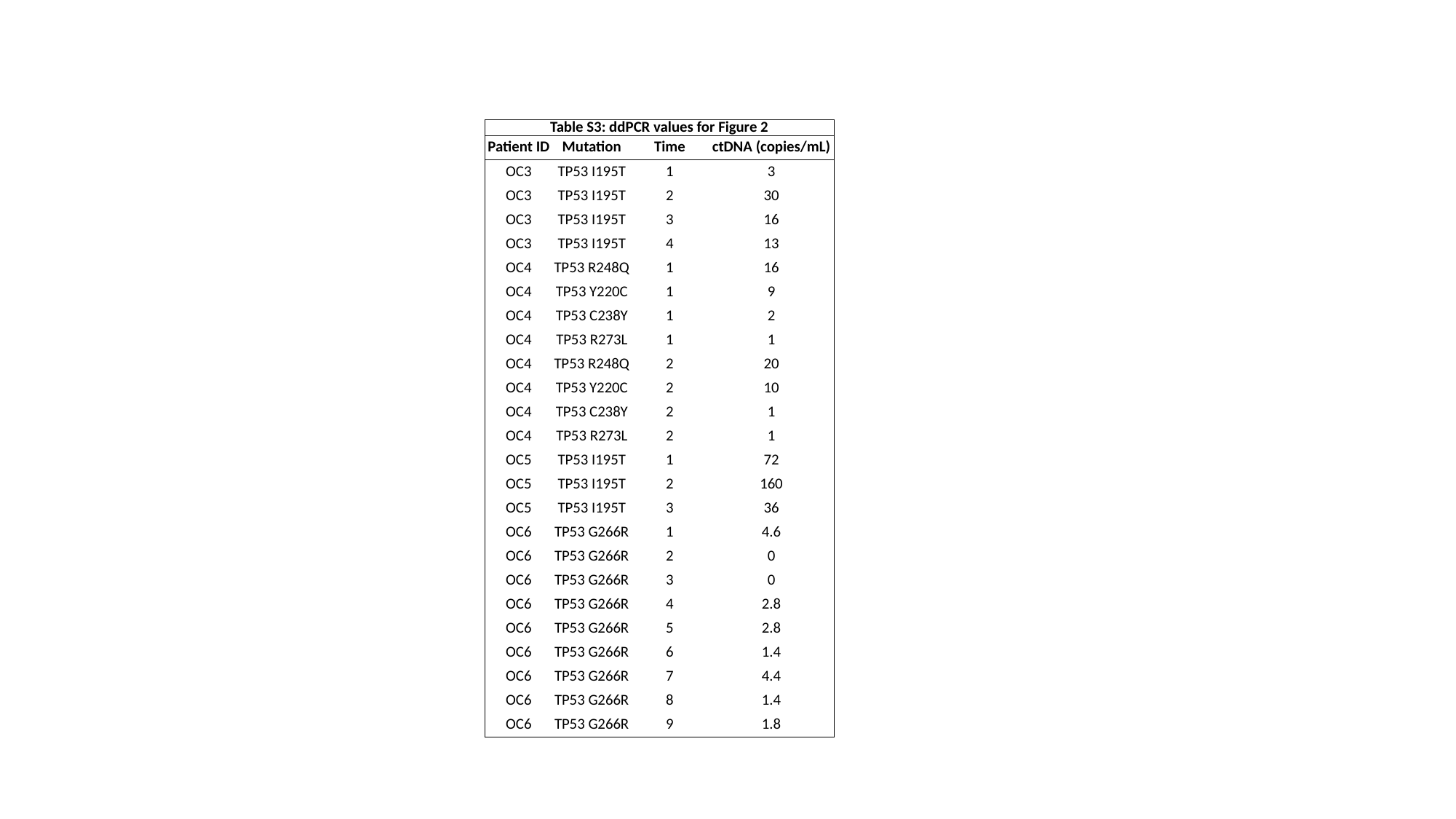

| Table S3: ddPCR values for Figure 2 | | | |
| --- | --- | --- | --- |
| Patient ID | Mutation | Time | ctDNA (copies/mL) |
| OC3 | TP53 I195T | 1 | 3 |
| OC3 | TP53 I195T | 2 | 30 |
| OC3 | TP53 I195T | 3 | 16 |
| OC3 | TP53 I195T | 4 | 13 |
| OC4 | TP53 R248Q | 1 | 16 |
| OC4 | TP53 Y220C | 1 | 9 |
| OC4 | TP53 C238Y | 1 | 2 |
| OC4 | TP53 R273L | 1 | 1 |
| OC4 | TP53 R248Q | 2 | 20 |
| OC4 | TP53 Y220C | 2 | 10 |
| OC4 | TP53 C238Y | 2 | 1 |
| OC4 | TP53 R273L | 2 | 1 |
| OC5 | TP53 I195T | 1 | 72 |
| OC5 | TP53 I195T | 2 | 160 |
| OC5 | TP53 I195T | 3 | 36 |
| OC6 | TP53 G266R | 1 | 4.6 |
| OC6 | TP53 G266R | 2 | 0 |
| OC6 | TP53 G266R | 3 | 0 |
| OC6 | TP53 G266R | 4 | 2.8 |
| OC6 | TP53 G266R | 5 | 2.8 |
| OC6 | TP53 G266R | 6 | 1.4 |
| OC6 | TP53 G266R | 7 | 4.4 |
| OC6 | TP53 G266R | 8 | 1.4 |
| OC6 | TP53 G266R | 9 | 1.8 |

## Slide 4
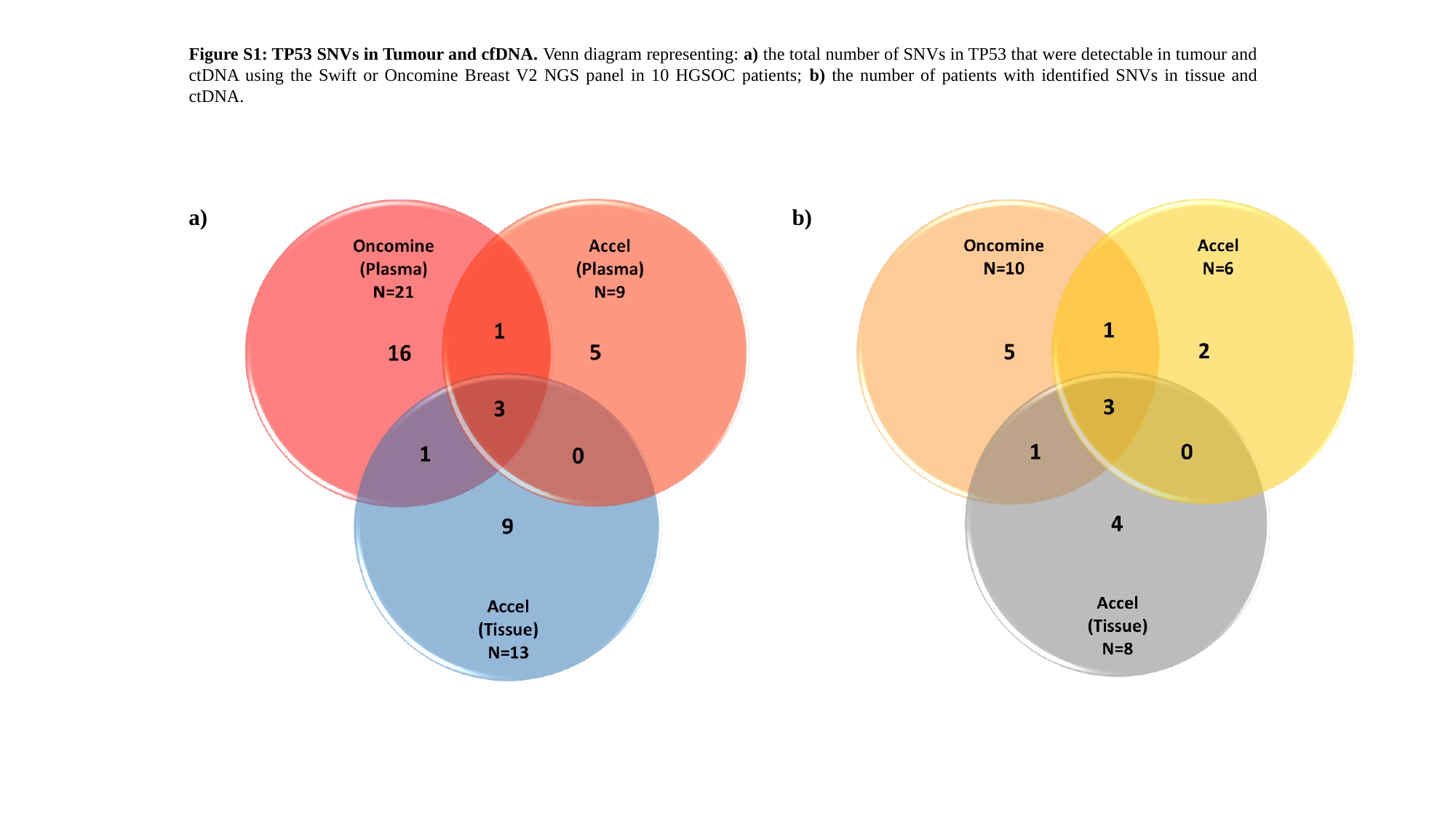

Figure S1: TP53 SNVs in Tumour and cfDNA. Venn diagram representing: a) the total number of SNVs in TP53 that were detectable in tumour and ctDNA using the Swift or Oncomine Breast V2 NGS panel in 10 HGSOC patients; b) the number of patients with identified SNVs in tissue and ctDNA.
a)
b)

## Slide 5
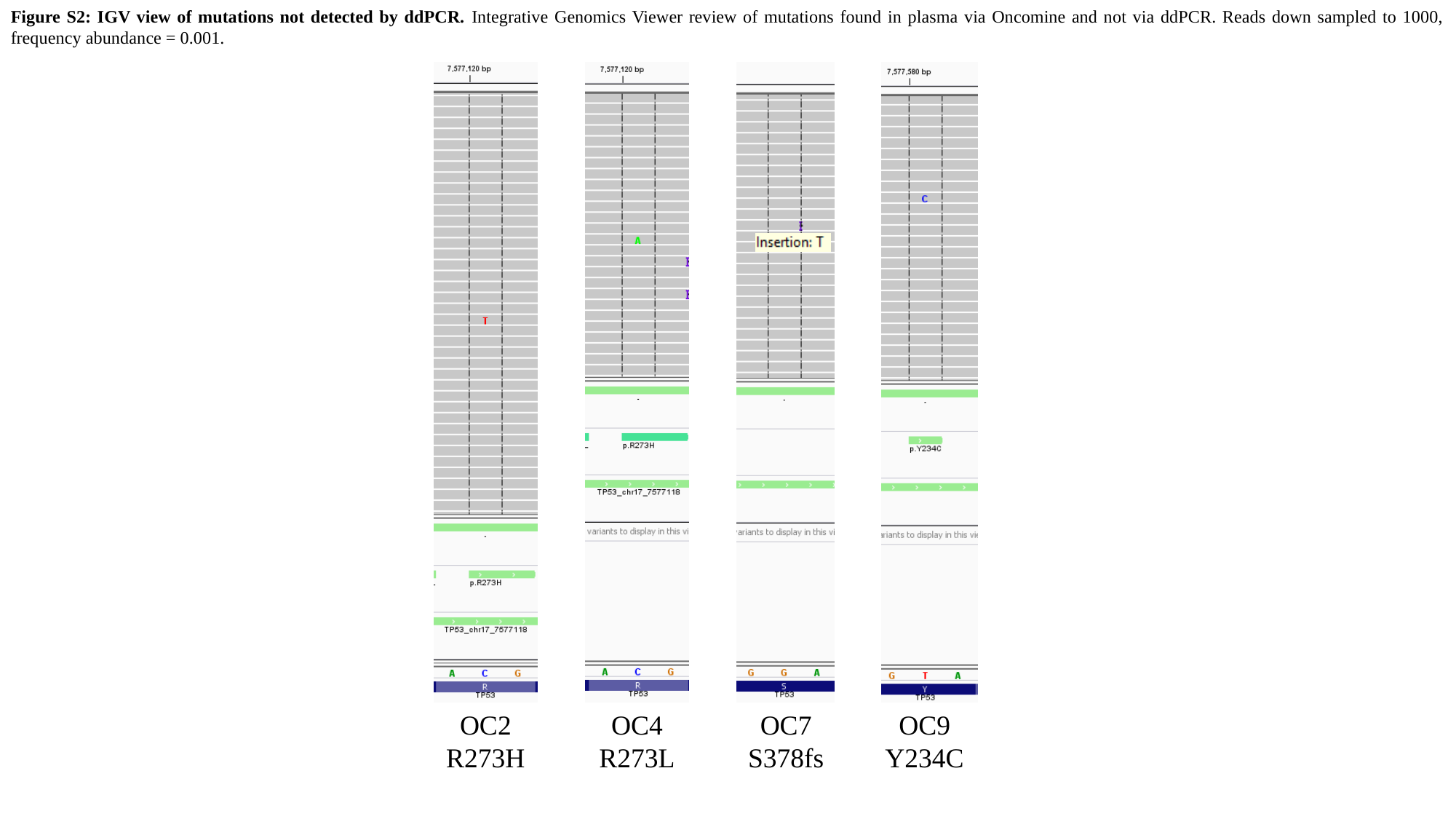

Figure S2: IGV view of mutations not detected by ddPCR. Integrative Genomics Viewer review of mutations found in plasma via Oncomine and not via ddPCR. Reads down sampled to 1000, frequency abundance = 0.001.
OC9
Y234C
OC2
R273H
OC4
R273L
OC7
S378fs
